# Congenital Pouch Colon with Rectal Atresia: A Rare Association

**DOI:** 10.21699/jns.v5i4.411

**Published:** 2016-10-10

**Authors:** Bilal Mirza, Muhammad Saleem

**Affiliations:** Department of Pediatric Surgery The Children's Hospital and the Institute of Child Health Lahore, Pakistan

**Dear Sir**

Rectal atresia is categorized in rare variants of anorectal malformations (ARM) and comprised of only 1-2% of all cases of ARM. Similarly, congenital pouch colon (CPC) is placed under the rare malformations category. Although it is scarce in prevalence in most part of the world, however, it forms 10-20% cases of ARM in south Asian countries like India, Pakistan, and Nepal. Concurrent presence of rectal atresia and CPC is exceedingly rare and only 4 cases have been described so far.[1-4] We are reporting another case and first from Pakistan, of this concurrence. 

A 2-day-old male neonate, weighing 2.5 kg, presented with failure to pass meconium since birth. On examination abdomen was distended but not tense or tender. Perineal examination showed normal buttocks and normally placed anus. A rectal examination with thermometer could not negotiate beyond 2-3 cm from anal verge. The invertogram, with contrast filled nelaton tube in the anus, showed a distance of about 2-3cm between proximal end of nelaton tube and the distal most dilated gas shadow (Fig. 1). Supine x-ray abdomen depicted air vesicogram besides dilated bowel shadows. A preliminary diagnosis of rectal atresia with rectourinary fistula was made. At operation, type IV congenital pouch colon was encountered (Fig. 1) which was excised with repair of colovesical fistula; and end colostomy was made. At the age of 3 years, abdomino-PSARP was done to anastomose mobilized end colon with the rectal pouch. Patient is doing fine on follow-up.

**Figure:**
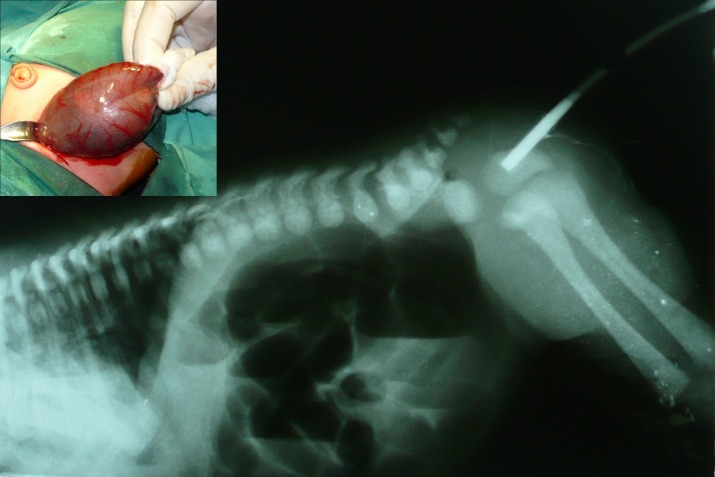
Figure 1: Invertogram showing a moderately dilated gas shadow in the pelvis at a distance of 2-3cm from contrast filled nelaton passed through the patent anus. Inset shows type 4 pouch colon.

Mathur et al, reported first case of this concurrence in 2002 followed by Kazez et al in 2009, Parelkar et al in 2010, and Singh et al in 2011. The diagnosis is suspected when a in a normally placed anus, rectal examination cannot negotiate thermometer beyond 2-3 cm from anal verge; and abdominal radiograph shows a big gas shadow in the abdomen with or without air vesicogram. Except the patients without colovesical fistula, there is meconuria may be present which may at times decompress the pouch colon and precludes its detection on abdominal roentgenogram. Majority cases of rectal atresia can eventually be managed with PSARP approach, however, in case of concomitant CPC, abdominal approach is always added to address CPC and associated colovesical fistula. The reported cases have been managed by primary abdomino-transanal pull through and staged abdomino-PSARP procedures. In case of staged approach, the pouch must be excised with ligation or repair of colovesical fistula during first stage with formation of end stoma as we did during initial surgery in neonatal life. In case of retention of pouch with big fistula various complications can arise; Singh et al reported hyperchloremic metabolic acidosis in their case where urine started collecting in the retained CPC through a big fistula.

## Footnotes

**Source of Support:** Nil

**Conflict of Interest:** None
